# The neostriatum: two entities, one structure?

**DOI:** 10.1007/s00429-015-1000-4

**Published:** 2015-02-05

**Authors:** Violeta G. Lopez-Huerta, Yoko Nakano, Johannes Bausenwein, Omar Jaidar, Michael Lazarus, Yoan Cherassse, Marianela Garcia-Munoz, Gordon Arbuthnott

**Affiliations:** 1 Brain Mechanisms for Behavior Unit, Okinawa Institute of Science and Technology Graduate University, 1919-1 Tancha, Onna-Son, Kunigami Gun, Okinawa, 904-0412 Japan; 2 University of Tsukuba, Ibaraki, 305-0006 Japan

**Keywords:** Adeno-associated virus, Limbic system, Reach-to-grasp, Neuromodulation, Volume transmission, Dopamine

## Abstract

The striosome (or patch) was first identified with anatomical techniques as neurons organized in a three-dimensional labyrinth inserted in and interdigitating the rest of neostriatum: the matrix. Striosome and matrix rapidly became known as two neuronal compartments expressing different biochemical markers, embryonic development and afferent and efferent connectivity. In spite of extensive intrinsic neuronal axonal and dendritic extensions supposed to exchange information between matrix and striosomes, evidence suggested the presence of independent areas. Here, we report that indeed these two areas do not exchange synaptic information. We used genetic expression of channel rhodopsin 2 carried by adeno-associated virus serotype 10 (AAVrh10) that only expresses in neurons of the matrix compartment. Whole-cell patch-clamp recordings of matrix neurons activated by light pulses consistently produced inhibitory postsynaptic currents (IPSCs), but the same manipulation did not evoke IPSCs in striosome neurons. The matrix contains both direct and indirect striatal output pathways. By targeting striatal matrix expression of designer receptors exclusively activated by a designer drug (DREADD) hM3di carried by AAVrh10, we were able to inhibit the matrix neuronal compartment of the dorsolateral striatum during performance of a learned single-pellet reach-to-grasp task. As expected, inhibition of matrix neurons by systemic administration of DREADD agonist clozapine-*n*-oxide interfered with performance of the learned task.

**Electronic supplementary material:**

The online version of this article (doi:10.1007/s00429-015-1000-4) contains supplementary material, which is available to authorized users.

## Introduction

Circumscribed “islands of dopamine terminals” scattered in striatum (Olson et al. 1972) became the matrix and striosome (or patch) compartments described in the literature later (Pert et al. 1976; Herkenham and Pert 1981; Graybiel and Ragsdale 1978). They were anatomical curiosities: a large area of neostriatum with neurons of high acetylcholine esterase activity (AChE) (matrix) containing zones poorly stained for AChE (striosomes). As research progressed, the two compartments were observed in primate and non-primate species (Graybiel and Ragsdale 1978; Herkenham and Pert 1981; Wilson et al. 1987) and striosomes became the three-dimensional serpentine tube or labyrinth with finger-like branches (Groves et al. 1988; Desban et al. 1993; Mikula et al. 2009; Manley et al. 1994; Breuer et al. 2005) distinguished by their exclusive label to µ opiate receptor ligands (e.g., MOR1 and naloxone) (Herkenham and Pert 1981) and high enkephalin-like immunoreactivity (Graybiel et al. 1981).

Neurons of the matrix compartment make up about 80 % of striatal volume (Johnston et al. 1990; Gimenez-Amaya and Graybiel 1991; Mikula et al. 2009) and are rich in calbindin (Gerfen et al. 1985; Kawaguchi et al. 1989; Liu and Graybiel 1992). These neurons develop and migrate later in development than neurons in striosomes (Graybiel et al. 1981; Gerfen et al. 1987a; van der Kooy and Fishell 1987). Inputs to matrix originate in sensory and motor cortical areas (mainly layer V) (Gerfen 1984; Donoghue and Herkenham 1986; Flaherty and Graybiel 1991, 1993; Kincaid and Wilson 1996), prefrontal areas (Eblen and Graybiel 1995), parafascicular and centromedial thalamic nuclei (Herkenham and Pert 1981; Ragsdale and Graybiel 1991) and dorsal substantia nigra compacta (Gerfen et al. 1987b; Prensa and Parent 2001). Outputs project in two directions: to substantia nigra pars reticulata (direct pathway) and to globus pallidus (indirect pathway) (Gerfen 1984; Gerfen et al. 1985; Graybiel et al. 1979; Jimenez-Castellanos and Graybiel 1989; Kawaguchi et al. 1990). Consistent with its input–output patterns, movement-related increases in neuronal activity observed in freely moving rats are located in the matrix compartment (Heidenreich et al. 1994; Brown et al. 2002; Trytek et al. 1996).

Neuronal development and dopamine innervation occur earlier in striosomes than matrix (Graybiel et al. 1981; Gerfen et al. 1987a; van der Kooy and Fishell 1987; Seiger and Olson 1973). Inputs to striosomes are from prelimbic cortex (layers Vb and VI) (Gerfen 1984; Donoghue and Herkenham 1986; Kincaid and Wilson 1996), motor and somatosensory areas (Gerfen 1989), midline thalamic nuclei (paraventricular and rhomboid) (Ragsdale and Graybiel 1991) and neurons located in ventral substantia nigra compacta and substantia nigra pars reticulata (Gerfen et al. 1987b; Prensa and Parent 2001; Tokuno et al. 2002).

Although characterized by their µ opioid receptors, striosome neurons also express among others dopamine D1 or D2 receptors (Ambrose et al. 2004; Georges et al. 1999; Guttenberg et al. 1996). Outputs from these neurons are to substantia nigra pars compacta (Gerfen et al. 1987b; Watabe-Uchida et al. 2012), to a densocellular zone also poorly stained for AChE (Jimenez-Castellanos and Graybiel 1989). Striosomes provide a reciprocal loop with substantia nigra compacta to control dopamine release and reward-related calculations (Crittenden and Graybiel 2011). Consistent with a reward-related function, striosome neurons release endogenous enkephalin in response to chocolate eating (DiFeliceantonio et al. 2012) and rats learn to self-stimulate when electrodes are located in striosomes (White and Hiroi 1998). Still related to a limbic influence on behavior, mice with a selective striosome lesion were not able to perform in a rotarod task that involves a combination of motor coordination and control of fear of falling (Lawhorn et al. 2009).

In spite of great advances, there are still very important questions to answer regarding matrix and striosome compartments: (1) Do neurons located in striosomes and matrix talk to each other? (2) Can animals perform a learned task with an inhibited matrix compartment? Fortuitously, we found that the adenosine-associated virus (AAV) serotype rh10 infects neurons in the matrix, but not in the striosome compartment. This finding allowed us genetic expression of channel rhodopsin 2 (ChR2) or targeted expression of designer receptors exclusively activated by a designer drug (DREADD) hM3di carried by AAVrh10. Using these techniques, our results suggest that “no” is the likely answer to both questions.

## Materials and methods

### Animals

All our experiments complied with guiding policies and principles for experimental procedures endorsed by the government of Japan and supervised by the local Animal Care and Use Committee.

We used the following mice strains: wild-type c57BL/6N mice, Swiss Webster bacterial artificial chromosome (BAC) transgenic mice D1-eGFP or D2-eGFP, and D1c-Cre transgenic lines. Animals were postnatal 25–30 days of either sex for electrophysiological experiments and males only for behavioral experiments.

### Adenovirus (AAV) functionally expressed in mouse striatum

We used the following adeno-associated virus (AAV): AAV1-dflox-hChR2-mCherry (Cre-activated, Addgene 20297, Penn Vector core), AAV10-Syn-ChR2-mCherry and AAV10-syn-hM3Di-mCherry (provided by Dr. Micheal Lazarus). In aseptic conditions and under isofluorane anesthesia (IsoFlo Abbot, Ill) animals received for electrophysiological experiments unilateral injections of a virus containing ChR-2 (0.3 μl) and for behavioral experiments bilateral injections of a virus containing hM3di (0.2 μl). Striatal stereotaxic coordinates were AP 0.98 mm, LM −1.89 mm, DV −3.45 mm for unilateral injections or AP 1.2 mm, LM 2.28, DV 3.35 for bilateral injections (Franklin and Paxinos 2008).

### Slice preparation

Sagittal slices (250 µm) were obtained from AAV-injected animals 2 weeks post-surgery to allow virus expression. Mice were anesthetized via isofluorane inhalation and perfused transcardially using cold saline containing (in mM): 124 choline chloride, 2.5 KCL, 2 MgCl_2_, 20 HEPES, 1.2 NaH_2_PO_4_·H_2_O, 1 CaCl_2_, 1 ascorbic acid, and 3 pyruvate and 10 glucose saturated with 95 % O_2_ and 5 % CO_2_, pH 7.4, 298 mOsm/l. Slices were cut and transferred to regular artificial cerebral spinal fluid containing the following in mM: 136 NaCl, 3.5 KCl, 1 MgCl_2_, 2.5 CaCl_2_, 26 NaHCO_3_ and 11 glucose saturated with 95 % O_2_ and 5 % CO_2_, where they remained for at least 1 h before recording at room temperature (21–25 °C).

### Electrophysiological recordings

We performed whole-cell patch-clamp recordings with borosilicate glass micropipettes (Harvard Apparatus 30-0057) heat polished to obtain direct current resistances of ∼4–6 MΩ. Micropipettes were filled with an internal solution containing in mM: 115 KH_2_PO_4_, 2 MgCl_2_, 10 HEPES, 0.5 EGTA, 0.2 Na_2_ATP, and 0.2 Na_3_GTP. The recordings were made with a microelectrode amplifier with bridge and voltage clamp modes of operation (BVC-700A, Dagan Co, Minneapolis, MN, USA). In some cases, conventional characterization of neurons was made in voltage and current clamp configurations. Access resistances were continuously monitored to be less than 20 MΩ, experiments with changes over 20 % were interrupted and terminated. Software designed in LabVIEW environment (National Instruments) was used for data acquisition and we performed analysis with Origin (version 8.6, Microcal, Northampton, MA, USA).

### Stimulation

Synaptic events were evoked by photostimulation using an optic fiber and LED driver and fiber-coupled LED light source (DC2100, OGKR2 Thorlabs, Newton, NJ, USA) Stimulation frequency was controlled with a computer interface to quickly adjust stimulus parameters during the experiment. Traces shown are the average of near 5 min recordings (25–30 traces) for a given condition.

In some experiments, pressure injection of glutamate (puff) (1 mM/20 psi/50 ms) was delivered via a Picopump (PV820 World Precision Instruments, Sarasota, FL, USA) through a pipette located close to a selected area. In every occasion, we controlled the extent of the glutamate puff by mixing Alexa 598 in the glutamate stock solution to adjust the diffusion to approximately 50 µm diameter fluorescent drop.

### Single-pellet reach-to-grasp task

Litters of mice bred at OIST animal facility were kept in a room with controlled environment (temperature: 21 ± 1 °C; humidity 55 %; light schedule 12/12 h with lights off at 7 p.m.) and were weaned at postnatal day 21. Weaned pups were housed in same-sex groups of 2–4. Standard rodent pellets and water were provided ad libitum except during test period (W5-postnatal day 26) when food restriction was introduced. We used a training box of the same dimensions and followed procedures as established by Marques and Olsson (2010). Briefly, food restriction schedule provided enough food to maintain approximately 85 % of body weight. We used as reward 20 mg dustless precision pellets with chocolate flavor (Bio-Serv, USA), during training and testing mice received 20 pellets in a 10-min daily session. Three days prior to test period, mice were habituated to reward pellets scattered on the bottom of the cage once daily (0.2 g/animal/day). Daily after training and testing, cage food was allowed. For training, mice were divided in two groups: sham (AAV1-ChR2-mCherry-injected animals) and hM3di group (AAV10-hM3di-mCherry-injected mice). Mice were observed from the front of the cage. Shaping of grasping response (from pellet retrieval with the tongue to use of a preferred paw) was performed on 10-min sessions for 2 days. The preferred paw was determined on the first 10 attempts to reach. If a mouse used both paws, the preferred paw was considered the one used more frequently (out of 10 reaches). Grasp response was made easier by gradually moving the pellet towards the indentation contralateral to the preferred paw (Miklyaeva et al. 1994). Two pre-training days were followed by five consecutive days of training, with daily sessions lasting until 20 pellets were successfully retrieved and eaten or a maximum of 10 min had elapsed. Starting on training day two, 2 h before training, mice were injected 200 µl of isotonic saline solution (i. p.) replaced on days 5, 6 and 7 by the inert hM3di-agonist clozapine-*n*-oxide (CNO, 3 mg/kg i.p.) (Armbruster et al. 2007; Lee et al. 2014). We recorded all sessions with a conventional camcorder for further analysis. Performance was analyzed according to the quantitative measures established by Marques and Olsson (2010): Latency to the first reaching attempt (the single first time the mouse performed a reaching movement), reaching accuracy = (number of pellets retrieved/number of reaches) × 100, latency to retrieve one pellet (the latency of the first successful grasp on each testing day) and time of performance = latency to retrieve 5 pellets − latency to retrieve 1 pellet.

### Drugs

CNQX (6-cyano-7-nitroquinoxaline-2,3-dione), gabazine (SR95531), lidocaine *N*-ethyl bromide (QX-314), glutamic acid (Sigma-Aldrich, San Luis, MO, USA) were prepared freshly in stock solutions and added to perfusion during experiments at the required concentration. Clozapine-*n*-oxide (CNO or 8-chloro-11-(4-methyl-1-piperazinyl)-5H-dibenzo[b,e](1,4)diazepine N-oxide (Sigma-Aldrich, San Luis, MO, USA) was also prepared fresh for systemic administration.

### Histology

Mice were intracardially perfused briefly with phosphate buffer 0.01 M (pH 7.4) followed by phosphate buffer containing 4 % paraformaldehyde and 14 % picric acid. Brains were postfixed for at least 2 h and then cryoprotected in a 50/50 mixture of fixative and 20 % sucrose in 0.01 M phosphate buffered saline (PBS). Sections were cut at 60 µm on a sledge microtome with a freezing stage (Yamato electrofreeze, MC-802A), washed in PBS and incubated in 20 % normal goat serum for 1 h. Primary antibodies to MOR1 (guinea pig polyclonal 1:1,000, Millipore, Darmstadt, Germany) were incubated overnight at 4 °C and stained with goat secondary antibodies. At least 2 h were allowed for binding before rinsing in PBS. Sections were mounted on slides; Vectamount AQ (Vector) was used to fix the coverslips. To inspect stained tissue a spinning disc confocal microscope (Olympus BX-DSU) and confocal microscope (Carl Zeiss LSM780) were used and pictures were taken using Neurolucida software or ZEN software and a Hamamatsu (EM-CCD C91) camera.

## Results

### Fortuitous discovery

**Fig. 1:**
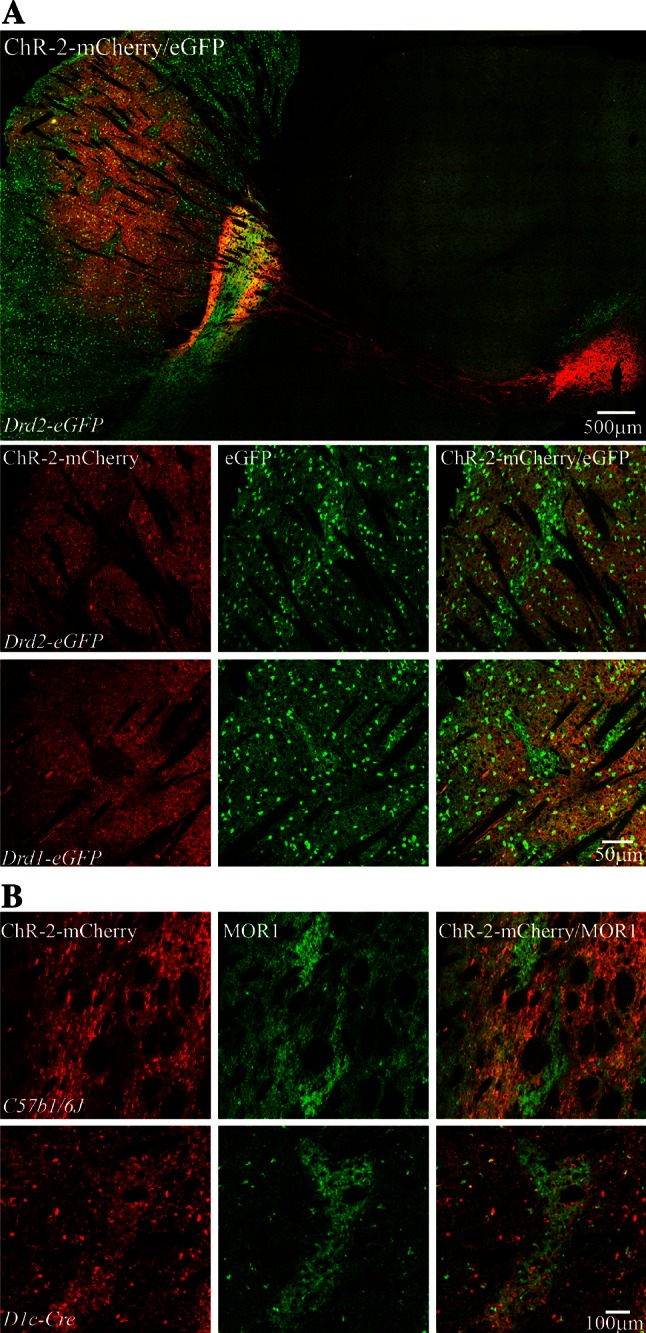
Typical striatal expression pattern of GFP and ChR2-mCherry in AAV10-injected Drd2-eGFP and Drd1-eGFP mice. **a** *Top* panoramic sagittal view of double immunofluorescence staining GFP (*green*) and ChR2-mCherry (*red*) in AAV10-injected (*Drd2-eGFP*) mouse brain. **a** *Bottom* representative pictures of double immunofluorescence staining to illustrate the distribution of ChR2-mCherry (*red*), GFP (*green*) and merged images for mice expressing Drd2-eGFP or Drd1-eGFP. **b** *Top* coronal sections of mouse striatum show the expression of MOR1 (*green*) and ChR2-mCherry (*red*) and their superimposition. The mCherry expression avoids the striosome in C57B16 mice. **b** *Bottom* immunofluorescence distribution of MOR1 (*green*) and ChR2-mCherry (*red*) carried by an injection of AAV1 in a D1-Cre mouse. The mCherry expression is present in both compartments

**Fig. 2:**
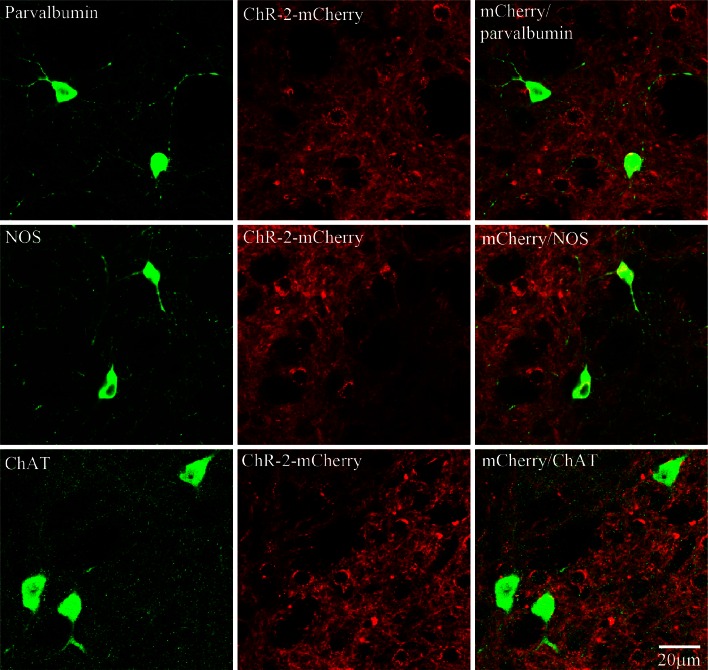
Striatal interneurons express ChR2-mCherry in AAV10-injected mice. Besides matrix neurons AAVrh10 virus also infected interneurons that express nitric oxide synthase (*NOS*), parvalbumin or choline acetyl transferase (*ChAT*). Viral presence is indicated by mCherry (*red*) in interneurons (*green*). In image superimpositions, the presence of virus is clearly seen in the cell bodies of interneurons. Due to the large size of cholinergic neurons, expression of mCherry only seen in the surrounding membrane—although present—is more difficult to ascertain

We injected striatum of wild-type or BAC D1 and D2 mice with AAVrh10 to express ChR2-mCherry under Syn promoter. With this manipulation areas of null ChR2-mCherry expression were embedded in large areas effectively infected resembling the matrix–striosome compartment organization (*n* = 21). Confirmation of the presence of such striosome–matrix arrangement in the head of striatum was obtained by immunohistochemistry. Non-infected ChR2-mCherry regions contained the signature staining of striosomes Mor1-positive and calbindin-negative neurons (Gerfen et al. 1985; Pert et al. 1976). Strikingly, independent of mouse strain or viral fusion proteins, the adenovirus serotype rh10 selectively targeted striatal neurons of the matrix compartment (Fig. 1). Striatal interneurons also expressed the AAVrh10 viral vector and probably account for the small 7 % of infected neurons sometimes observed in striosome compartment. Some optical slices, however, were difficult to discern with absolute certitude (Fig. 2).

### Is synaptic information transferred between striosome and matrix compartments?

Visualization of mCherry allowed identification of striatal compartments: matrix compartment as mCherry (+) and striosome compartment as mCherry (−). Accordingly, neurons from either compartment were selected for patch-clamp recording in current and voltage clamp modalities and later their compartment membership confirmed by post hoc immunostaining of Alexa-488 filled neurons (*n* = 8).

**Fig. 3:**
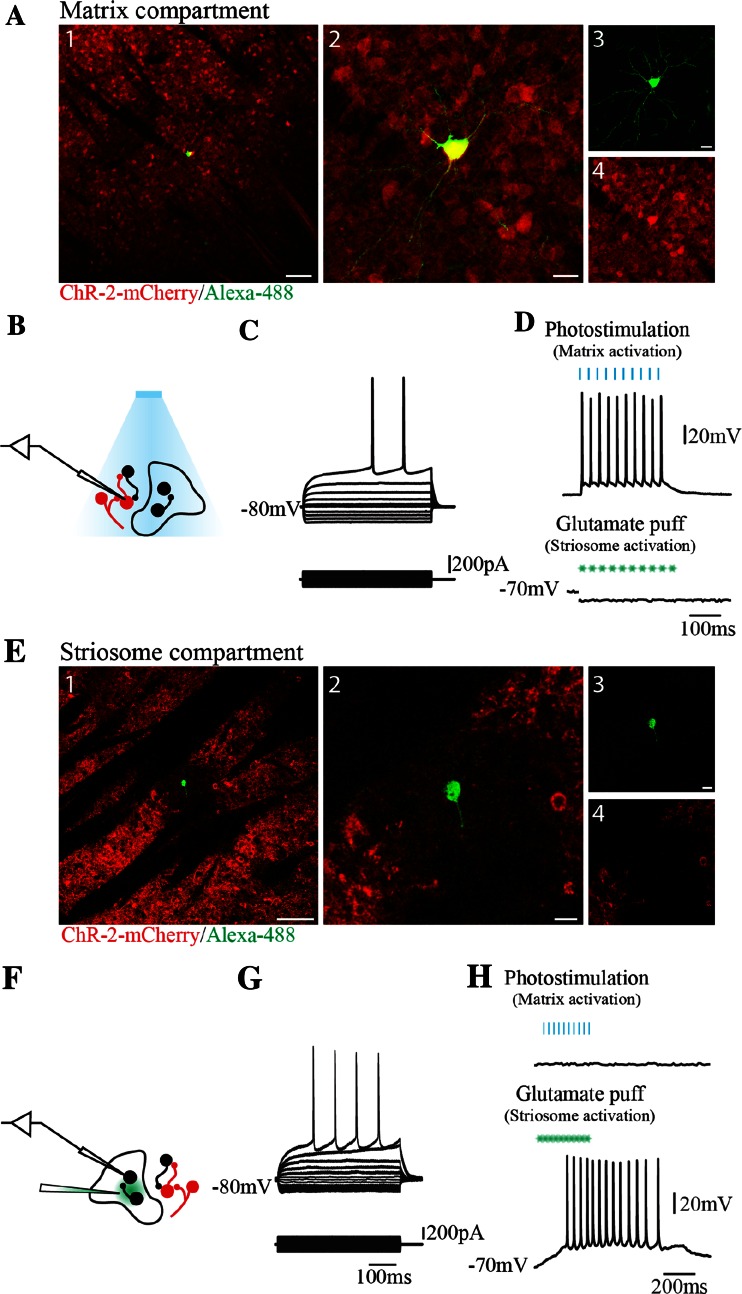
Targeted electrophysiological recordings from matrix and striosome compartments. **a** Striatal spiny neuron (SPN) filled during recording (Alexa-488/Biocytin, *green*) located in the matrix compartment (ChR2-mCherry, *red*). *1* Merged image of Alexa-488 (*green*) and ChR2-mCherry (*red*), *2* zoom of *1*, *3* Alexa-488 only, *4* ChR2-mCherry only. **b** Schematic representation of stimulation and recording protocol. **c** Representative firing patterns of SPN in the matrix compartment. Voltage responses to hyperpolarizing and depolarizing current injections of neuron shown in **a**. **d** Action potentials evoked by photostimulation of ChR2. **e** SPN filled during recording (Alexa-488/Biocytin, *green*) located in the striosome compartment (ChR2-mCherry, *red*). *1* Merged image of Alexa-488 (*green*) and ChR2-mCherry (*red*), *2* Zoom, 3 Alexa-488, 4 ChR2-mCherry. **f** Schematic representation of stimulation and recording protocol. **g** Representative firing patterns of SPN in the striosome compartment. Voltage responses to hyperpolarizing and depolarizing current injections of neuron shown in **e**. **h** Action potentials evoked by delivery of a glutamate puff (1 mM)

In the matrix compartment, 97 % percent of recorded cells expressed ChR2-mCherry (*n* = 34). As illustrated in Fig. 3 (top) their illumination produced consistent action potential firing at frequencies less than 40 Hz (Boyden et al. 2005; Zhang et al. 2006) and a glutamate puff (1 mM) excitation of nearby striosome neurons did not influence matrix cells. The striosome compartment (Fig. 3 bottom) was characterized by lack of ChR2-mCherry expression (*n* = 11). Photostimulation, therefore, did not depolarize neurons in this compartment although action potentials were elicited by delivery of a localized glutamate puff close to the recorded cell. Glutamate-evoked current had a reversal potential around 10 mV (Keller et al. 1991) (*n* = 4, Online Resource 1).

### Is there synaptic connectivity between striosome and matrix compartments?

**Fig. 4:**
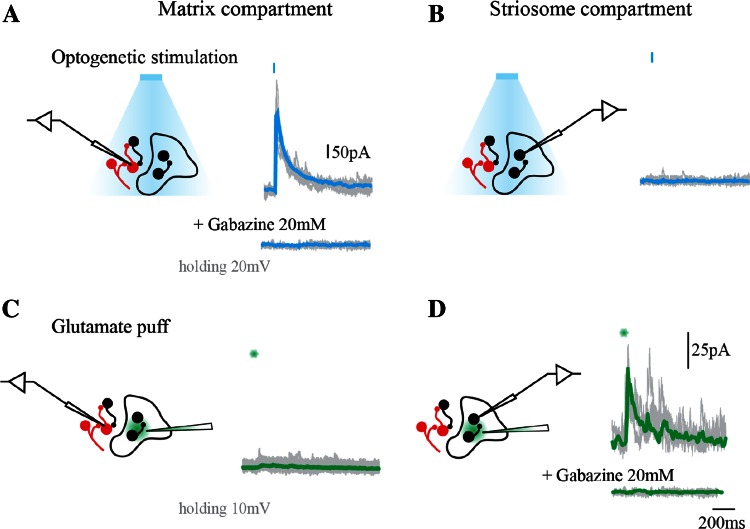
Matrix and striosome compartments do not share synaptic connectivity. **a** Evoked IPSC in a neuron of the matrix compartment induced by photostimulation (holding potential 20 mV). Trace below in the presence of GABA_A_ antagonist gabazine (20 µM). *Blue bars* indicate time of stimulation. **b** Voltage trace of a recorded neuron located in the striosome compartment during photoactivation of neighboring matrix area. **c** Voltage trace of a recorded neuron from matrix compartment during activation by glutamate puff of neighboring striosome. **d** Evoked IPSC in a striosomal neuron by activation of surrounding striosome with a glutamate puff. Trace below in presence of gabazine (20 µM)

Combined recordings of neurons located either in matrix or striosome compartments with a—global activation of striatal matrix neurons expressing channel rhodopsin and b—circumscribed neuronal activation by delivery of glutamate, indicated that compartments do not share synaptic connectivity. As illustrated in Fig. 4, neurons in the matrix compartment recorded in voltage clamp mode and briefly photostimulated (2–5 ms pulse) responded with inhibitory postsynaptic currents (IPSCs) with an average amplitude of 542.7 ± 292.1 pA, *n* = 11.

To avoid contamination, all IPSCs were recorded at 20 mV (photocurrent reversal potential, Online Resource 2). Complete blockade of IPSCs with the GABA_A_ antagonist gabazine (20 µM) confirmed the GABAergic origin of recorded currents. No responses from matrix neurons were observed if a glutamate puff was delivered to the striosome compartment (*n* = 4). Neurons in striosomal compartment recorded in voltage clamp mode and briefly photostimulated (2–5 ms pulse) did not evoke IPSCs at any holding potential even when located near compartment boundaries (*n* = 7), however, a brief puff of glutamate inside striosome compartment reliably evoked IPSCs with an average amplitude of 70.6 ± 24.3 pA, *n* = 6 followed by asynchronous IPSCs afterwards. Blockade of IPSCs with gabazine (20 µM) again confirmed the GABAergic origin of IPSCs.

### Selective inhibition of matrix compartment impairs skilled motor behavior

**Fig. 5:**
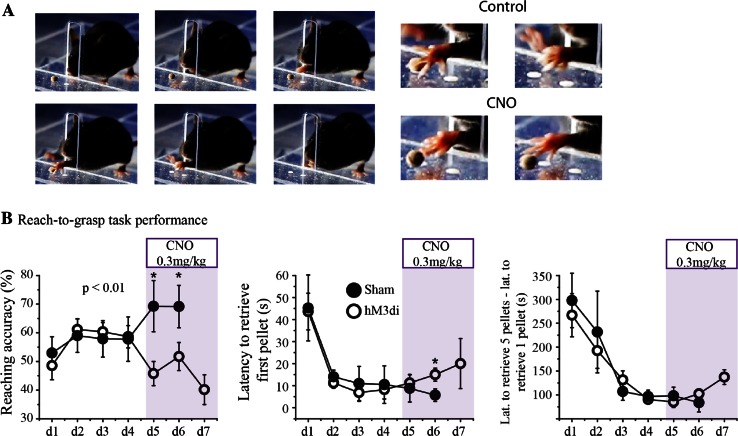
Performance of a single-pellet reach-to-grasp task following neuronal inhibition of the matrix compartment. **a** Photographs of a mouse performing a successful reach-to-grasp trial. **b**. Quantitative evaluation of mice performance for sham and hM3di group in terms of reaching accuracy (successful trials/total trials × 100), latency for first pellet retrieval and latency to retrieve 5 pellets—latency to retrieve first pellet

We trained mice in a single-pellet reach-to-grasp task to evaluate skilled motor performance during selective inhibition of the dorsolateral striatal matrix compartment. We used targeted expression of designer receptors exclusively activated by a designer drug (DREADD) hM3di carried by AAV serotype 10 in dorsolateral striatum. Of 28 mice, only 15 (53.6 %) learned the task and expressed virus successfully, of those 7 mice were AAV1-ChR2-mCherry-injected mice (sham group) and 8 were AAV10-Syn-hM3di-injected mice (hM3di group). The right forepaw was preferred by 60 % of the animals. As described in methods mice were expected to reach and grasp 20 pellets in a 10-min daily session. Figure 5 shows performance rates of sham and hM3di groups as reaching accuracy, latency to retrieve first pellet and latency to retrieve 5 pellets—latency to retrieve first pellet (Marques and Olsson 2010). The learning curve of both groups was similar, mice increased reaching accuracy through sessions up to 57 ± 6 % and decreased task time to 96 ± 7 s. In sessions where DREADD agonist clozapine-*n*-oxide (CNO, 3 mg/kg, i. p.) was administered to animals of both groups, a significant difference in reaching accuracy between groups was observed. Inhibition of matrix neurons by CNO administration resulted in low reaching accuracy (46 ± 5 % for hM3di and 69 ± 7 % for sham group). All mice showed similar motivation to perform the task taking equal amount of time during training sessions as shown by the time required to collect 5 pellets minus the latency of the first successful grasp of the day.

## Discussion

Our experiments demonstrate that AAV serotype rh10 expresses in striatal neurons located in matrix compartment sparing 93 % of neurons in striosomes. With functional viral expression of AAVrh10, we specifically targeted the matrix compartment to express ChR2-mCherry fusion proteins. This procedure allowed directed patch-clamp recordings of neurons belonging to both areas and selective photoactivation of matrix compartment. We observed absence of physiological synaptic connections to neurons in striosomes from neurons in matrix (Figs. 3, 4). In addition, we performed selective matrix inhibition on a skilled motor task by expression DREADD hM3di, which led to decreased performance. Taken together our results using a new tool to selectively manipulate the matrix compartment demonstrate lack of functional connectivity between striosome and matrix and confirm the participation of matrix and dorsolateral striatum in acquired motor skills.

### Adeno-associated virus vectors

Adeno-associated viruses are single-stranded DNA-containing viruses of 57 serotypes classified in 7 species (Chen and Lee 2014). Six types of adenoviruses (AAV1-6) have been isolated from primates (AAV1), non-human primates (AAV2-3) and humans with an 80 % homology (Xiao et al. 1999). Although isolation of an AAV2 clone facilitated development of stable vectors, the level of gene expression was low. Since proteins that determine cell entry present in capsid—protein shell or viral envelope—are directly related to gene expression (Cearley et al. 2008), successful attempts to engineer serotypes using AAV2 while modifying cell entry pathways (Ellis et al. 2013; Rabinowitz et al. 2004; Choi et al. 2005) have resulted in more than 100 different AAV capsids cloned from primates and many others engineered (Surace and Auricchio 2008). Primary cell entry receptors are CD46, coxsackie virus and adenovirus receptor (CAR) and sialic acid. We used AAV1 (Vector systems, Phyl., PA, USA) and AAVrh10 (Inutsuka et al. 2014; Lazarus et al. 2011) and found that striatal neurons in matrix and striosome compartments get infected with AAV1, but AAVrh10 infects only neurons of matrix. Since changing promoter from synapsin to EF1α did not affect viral expression, it is possible that the entry receptor is responsible for differences we observed. The primary receptor for AAV1 is sialic acid (Ellis et al. 2013), but receptor of reengineered rhesus monkeys capsid of AAVrh10 is not known (Giove et al. 2010; Arnberg 2012). Since AAV1 and AAVrh10 used the same promoter and AAV2 backbone, it is possible that neuronal entry receptor density in striosome could be very low or other factors involving intracellular traffic to the nucleus, decapsidation or conversion of single-stranded DNA genome to double-stranded DNA could be participating (Ding et al. 2005).

It is interesting to point out that neurons belonging to striosome compartment, but not matrix, express limbic-associated membrane protein (LAMP) now called limbic system-associated membrane protein (LSAMP) (Cote et al. 1995; Chesselet et al. 1991). LSAMP is a cell surface glycoprotein important for neuronal adhesion and regional identity during development and in striosome formation. Importantly, LSAMP belongs to the immunoglobulin (Ig) protein superfamily with three Ig domains and a glycosylphosphatidylinositol anchor (Pimenta and Levitt 2004). Could it be that presence of this protein prevents entrance of AAVrh10 into striosome neurons?

The preference for AAVrh10 use in mice striata (Cearley and Wolfe 2006; Swain et al. 2014; Klein et al. 2008) should be of concern considering expression of virus located in the matrix.

### Is there a functional regulation between striosome and matrix compartments?

Anatomical evidence has shown that matrix and striosome neurons confine their axonal and dendritic processes to their own compartments (Penny et al. 1988; Kawaguchi et al. 1989; Graybiel et al. 1986; Bolam et al. 1988). Although interneurons sometimes appear to send a few dendrites across boundaries (Cowan et al. 1990; Penny et al. 1988; Chesselet and Graybiel 1986; Walker and Graybiel 1993; Walker et al. 1993; Kawaguchi 1992) and the notion of interneuron information sharing is appealing (Crittenden and Graybiel 2011), the question remains, however, as to the function of those neuronal processes. More behavioral and pharmacological studies are also needed since, so far, evidence of shared functions is not available. For instance, after comparison between compartments, striosomes display lower dopamine uptake sites (Graybiel and Moratalla 1989) and higher induction of early genes following systemic administration of cocaine, amphetamine and apomorphine (Canales and Graybiel 2000). Moreover, greater deterioration of motor performance, higher expression of fear and susceptibility to reinforcement follow impairment of mGlu1/5 signaling (Tappe and Kuner 2006). Increases in movement-related neuronal activity in matrix compartment have been observed in freely moving rats (Heidenreich et al. 1994; Brown et al. 2002; Trytek et al. 1996) consistent with the presence of both striatal direct and indirect pathways in the matrix compartment (Gerfen 1984; Gerfen et al. 1985; Graybiel et al. 1979; Jimenez-Castellanos and Graybiel 1989; Kawaguchi et al. 1990).

Our electrophysiological evidence confirms that matrix and striosome compartments do not share direct synaptic connections. AAV expression under synapsin promoter drove neuron-specific gene transfer in all neuronal types of matrix compartment (Figs. 1, 3). Through neuronal expression of fluorophore mCherry, we identified striosome and matrix compartments and avoided low probability of pair recordings (Tunstall et al. 2002) by delivering a localized glutamate puff restrained to a small area. No physiological evidence of inhibitory synaptic connectivity between compartments was observed, although we observed synaptic responses among neurons belonging to each compartment alone (Fig. 4).

In neurons expressing ChR2-mCherry, we recorded IPSCs at photocurrent reversal potential (20 mV). Simultaneous recording and photoactivation of the matrix compartment evoked robust inhibitory responses with amplitudes and success rate that suggests widespread neuronal recruitment (Chuhma et al. 2011; Tepper et al. 2008) (Fig. 4). Photoactivation of matrix while recording striosome neurons produced no physiological response. To verify neuronal viability, we also delivered a glutamate puff, depolarized the surrounding striosomal compartment and observed inhibitory synaptic responses. These striosome responses were of smaller amplitude than those evoked by photostimulation of the matrix, most likely due to a smaller number of stimulated neurons in the 50 µm area of glutamate diffusion (see “Materials and Methods”). To further seek compartmental connectivity, we recorded matrix neurons while stimulating adjacent striosomes by glutamate pressure ejection. Once more, we could not evoke any inhibitory response. The possibility of very long processes mediating striosome to matrix compartment connections cannot be ruled out with such circumscribed depolarizing effects. Thus, we cannot say with certainty that matrix and striosomes do not interact, but how communication happens remains a question.

### The matrix compartment is involved in expression of a learned reach-to-grasp skill

Striatal participation in movement and acquisition of motor skills has been importantly related to dopamine function. There is a direct correlation between the extent of striatal dopamine loss and gross impairment of motor function. Loss of skilled behavior is observed in the contralateral forepaw following unilateral dopamine depletion (Hamilton et al. 1985; Miklyaeva et al. 1994; Uguru-Okorie and Arbuthnott 1981; Vergara-Aragon et al. 2003; Whishaw et al. 1986).

Several attempts at dividing striatum in sensorimotor, associative and limbic areas have been proposed (Graybiel 2008). Conveniently, also afferents to striosome and matrix separate compartments in limbic and sensorimotor systems, respectively. Striosomes have been associated with limbic-related goal-directed behaviors difficult to interpret such as motivation, pleasure and fear (DiFeliceantonio et al. 2012; Tappe and Kuner 2006; White and Hiroi 1998) also closely related to dopamine function. Since the largest of compartments, the matrix, contains main outputs to both globus pallidus and substantia nigra, it is expected to participate in behaviors associated with striatal and corticostriatal function such skill procedural learning and habit formation. Consistently, in freely moving animals increased neuronal activity associated to movement is located in the matrix (Heidenreich et al. 1994; Brown et al. 2002; Trytek et al. 1996).

In our behavioral experiments, we aimed at the matrix compartment located in dorsolateral striatum associated with skill procedural learning (Balleine and O’Doherty 2010; Yin et al. 2006) and forepaw use (MacLellan et al. 2006; Aldridge et al. 2004; Pisa and Schranz 1988).

Specific expression of AAVrh10 in neurons belonging to matrix compartment allowed us to perform some preliminary behavioral experiments. We expressed DREADD-hM3di receptors that can be easily activated by CNO to cause long-lasting neuronal inhibition. With matrix neurons inactivated in dorsolateral striatum, mice lost the already proficient skill to reach and grasp a single chocolate pellet (Fig. 5). Motivation to perform was not altered since animals kept trying and eventually managed to collect 5 pellets in the same time as controls.

### What could be the function of an independent striatal striosome system?

In spite of how clear and important our results are, we are faced with more questions than answers: Does the striosome compartment represent a third striatal output pathway? Do striosomes modulate: motivational aspects and reinforcement? Aversive-related dopamine release? Humoral signals?

The feedback loop established between striosomes and substantia nigra compacta has already prompted the idea of a third output pathway originating in striosomes relevant in levodopa-induced dyskinesias (Graybiel et al. 2000). Striosome neurons could regulate dopamine release to maintain required longer lasting extracellular levels of striatal dopamine. Moreover, dopamine spillover and dopamine receptors located extrasynaptically (Cragg and Rice 2004; Yung et al. 1995) could influence the effect of dopamine on striatal population activity through volume transmission. Extracellular dopamine levels induced by regular dopamine firing could buildup slowly and modulate readout of incoming signals and reset neuronal excitability (Agnati et al. 2000). A dopaminergic tone on a long time scale has been associated with behavioral states such as hunger, satiation, uncertainty, punishment, aggression, fatigue and sleepiness (Schultz 2007a, b).

Although striosome output is directed to substantia nigra pars compacta some axons leave collaterals in globus pallidus, entopeduncular nucleus and to substantia nigra pars reticulata (Fujiyama et al. 2011). These outputs could provide an efferent copy of an ongoing selection from action programs already proven successful (or rewarded) in the animal’s repertoire (Fee 2012, 2014; Mink 1996; Redgrave et al. 1999).

Neuromodulators other than dopamine could also influence volume transmission in striosomes. The greater vascularity of this compartment compared to matrix (Breuer et al. 2005) might allow blood-borne humoral factors to enter striosome compartment and perhaps together with dopamine modulate reward signals and behavior. For instance, leptin, the fat-derived hormone that can override satiety signals and promote overeating, has been found to modulate basal ganglia activity (Farooqi et al. 2007). Striosomes could play a pivotal role in aspects central to learning theory by linking behavior with a future predictive value. Striosomes can directly influence dopamine release in response to limbic and also possibly blood-borne neuroendocrine inputs associated for instance to relief of pain, anxiety, thirst and hunger or even pair bonding.

## Conclusion

Differential in vitro stimulation and recording of neurons belonging to either striatal striosome or matrix compartments was possible, thanks to expression of AAVrh10 in matrix neurons. Neuronal synthesis of channel rhodopsin 2 (ChR2) and targeted expression of designer receptors exclusively activated by a designer drug (DREADD) hM3di carried by AAVrh10 allowed us to report that striatal and matrix compartments to not talk to each other via direct monosynaptic connections and that inactivation of the dorsolateral striatal matrix impairs performance of a reach and grasp skill. Alternative ways of communication between striatal compartments such as longer axonal pathways, and volume transmission are possible.

## Electronic supplementary material

Below is the link to the electronic supplementary material.

Supplementary material 1 (DOCX 189 kb)
