# Supplementary material for: The neostriatum: two entities, one structure?
Source: Brain Struct Funct. 2015 Feb 5;221(3):1737–49. doi: 10.1007/s00429-015-1000-4 (PMC4819794; doi:10.1007/s00429-015-1000-4)
Supplement: Supplementary file 1 — Supplementary material 1 (DOCX 189 kb) [file 429_2015_1000_MOESM1_ESM.docx]

THE NEOSTRIATUM: TWO ENTITIES, ONE STRUCTURE?

*Violeta G. Lopez-Huerta, Yoko Nakano, Johannes Bausenwein, Omar Jaidar, Michael Lazarus^1^, Y. Cherassse^1^, Marianela Garcia-Munoz and Gordon Arbuthnott*

Corresponding author:

Prof. Gordon Arbuthnott

email: [gordon@oist.jp](mailto:gordon@oist.jp)

postal addresss:

Okinawa Institute of Science and Technology Graduate University

1919-1 Tancha, Onna-Son

Kunigami Gun, Okinawa

904-0412 Japan

Telephone number: + 81-98-966-8402


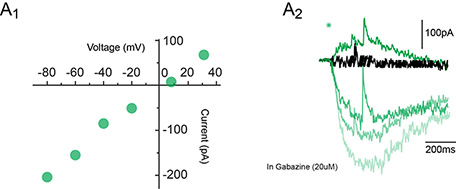


**Online Resource 1.** Glutamate-evoked current reversal potential (n=4).

**
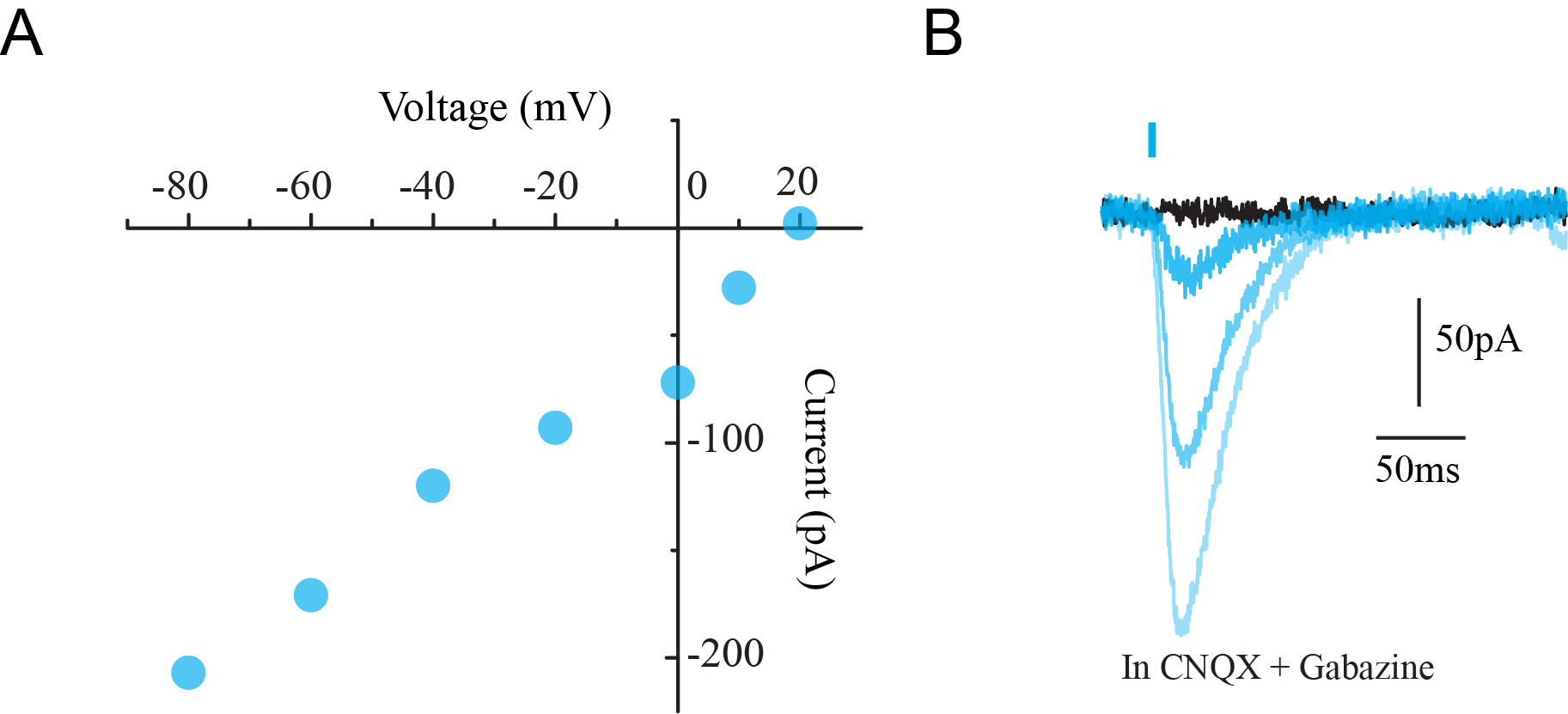
**

**Online Resource 2.** Photocurrent-evoked current reversal potential (n= 11).
